# Expression of the microtubule-associated protein 2 (MAP2) as a potential independent prognostic marker in prostate cancer

**DOI:** 10.1007/s00432-023-05579-0

**Published:** 2024-02-04

**Authors:** Johannes Stein, Eliana Krappe, Anika Kremer, Marcus V. Cronauer, Markus Essler, Alexander Cox, Niklas Klümper, Philipp Krausewitz, Jörg Ellinger, Manuel Ritter, Glen Kristiansen, Michael Majores

**Affiliations:** 1 Department of Urology, University Hospital Bonn, Venusberg Campus 1, 53127 Bonn, Germany; 2 Institute of Pathology, University Hospital Bonn, Venusberg Campus 1, 53127 Bonn, Germany; 3 Department of Nuclear Medicine, University Hospital Bonn, Venusberg Campus 1, 53127 Bonn, Germany

**Keywords:** MAP2, Prostate carcinoma, Prostatic intraepithelial neoplasia

## Abstract

**Purpose:**

Investigation of Microtubuli-associated Protein 2 (MAP2) expression and its clinical relevance in prostate cancer.

**Material and Methods:**

MAP2 expression was immunohistochemically analysed on radical prostatectomy specimens using whole block sections (*n* = 107) and tissue microarrays (TMA; *n* = 310). The staining intensity was evaluated for carcinoma, benign tissue and prostatic intraepithelial neoplasia. Expression data were correlated with clinicopathological parameters and biochemical recurrence-free survival. Additionally, MAP2 protein expression was quantitatively analysed in the serum of histologically confirmed prostate carcinoma patients and the control group using a commercial enzyme-linked immunosorbent assay.

**Results:**

MAP2 staining was significantly stronger in neoplastic tissue than in non-neoplastic prostatic glands, both in whole block sections (*p* < 0.01) and in TMA sections (*p* < 0.05). TMA data revealed significantly stronger MAP2 staining in high-grade tumors. Survival analysis showed a significant correlation between strong MAP2 staining in carcinoma and shortened biochemical recurrence-free survival after prostatectomy (*p* < 0.001). Multivariate Cox regression analysis confirmed MAP2 as an independent predictor for an unfavourable course. Mean MAP2 serum levels for non-PCA vs. PCA patients differed significantly (non-PCA = 164.7 pg/ml vs. PCA = 242.5 pg/ml, *p* < 0.001).

**Conclusion:**

The present data support MAP2 as a novel biomarker in PCA specimens. MAP2 is correlated with tumor grade and MAP2 high-expressing PCA is associated with an increased risk of biochemical recurrence after radical prostatectomy. Future studies are necessary to evaluate MAP2 as a valuable immunohistochemical biomarker in preoperative PCA diagnostic procedures, in particular with regard to treatment modalities.

## Introduction

Prostate cancer (PCA) is the second most frequently diagnosed cancer and the fifth leading cause of death from cancer in men worldwide (Sung et al. 2021). PCA is characterized by a variable biological behavior ranging from indolent to highly aggressive tumors with rapid disease progression. Early diagnosed and treated PCA has a good prognosis and is potentially curable. Serum prostate-specific antigen (PSA) is still the leading biomarker for the detection and follow-up management of patients with PCA. However, the sensitivity and specificity of PSA is limited, raising the risk of a false-positive or false-negative diagnosis. Moreover, PSA is unable to discriminate indolent from aggressive forms as high rates of overtreatment underline (Schröder et al. 2009). Diverse histological and cytological diagnostic schemes have been developed for an accurate assessment and grading of PCA. The Gleason Grading System is one of the most powerful prognostic evaluation criteria in PCA (Gleason 1966). The latest amendment of the Gleason Grading System was performed at the 2019 ISUP Consensus Conference (van Leenders et al. 2020). However, Gleason Grade is not sufficient to predict clinical progression accurately. In particular, patient selection for active surveillance is still a matter of debate. There has been controversy in recent years as to whether patients with a Gleason Score 7 (ISUP 2–3) may receive active surveillance. In the past, active surveillance was limited to patients classified into D’Amico low risk (implicates ISUP 1). Recently, results from the DETECTIVE trial suggested the inclusion of ISUP 2 and PSA < 10 ng/mL, clinical stage [< cT2a], and a low number of positive cores(Lam et al. 2019). However, the transitions from ISUP 2 to ISUP 3 are fluid, especially at the biopsy level with limited tissue for pathologic examination. Thus, the identification of biomarkers associated with aggressive phenotypes could help to improve the selection of the appropriate therapy.

Recently, aberrant expression of the microtubule-associated protein tau (MAPT) has been observed as an independent prognostic feature in PCA (Schroeder et al. 2019). MAPT was absent in normal prostate epithelial cells but detectable in a proportion of PCA cases and MAPT expression was associated with high Gleason Grade and earlier biochemical recurrence (BCR) (Schroeder et al. 2019). MAPT has also been associated with resistance to docetaxel in PCA cell lines (Yang et al. 2017). These findings indicate a possible role of microtubule-associated proteins in the pathogenesis and prognostic evaluation of PCA.

In the present study, the protein expression of the closely related Microtubule-associated Protein 2 (MAP2) in PCA was analysed in radical prostatectomy specimens, both in invasive tumors and its precursor lesions, i.e. prostate-intraepithelial neoplasia (PIN). MAP2 is another member of the Microtubule-associated proteins (MAPs) of the MAP2/Tau family, which includes the vertebrate proteins MAP2, MAP4, and Tau and homologs in other animals. Various upstream kinases and interacting proteins have been identified that regulate the microtubule-stabilizing activity of MAP2/Tau family proteins (Dehmelt and Halpain 2005). MAP2 plays a crucial role in the nucleation and stabilization of microtubules, promotion of tubulin polymerization, organelle transport as well as the anchorage of regulatory proteins (e.g. protein kinases) (Sánchez et al. 2000). Due to its regulatory functions, i.e. alteration of the phosphorylation state of cytoskeletal proteins, a possible role for tumor initiation and progression may be hypothesized.

MAP2 expression has been observed in various tissues with neuroendocrine features such as Merkel cell carcinomas or small cell lung cell cancer (Liu et al. 2003b, a; Zhou et al. 2013). Furthermore, it may be found in melanocytic lesions, i.e. melanocytic naevi and melanomas (Fang et al. 2001; Gambichler et al. 2009). In recent studies, MAP2 expression was detected in gastric, breast and pancreatic cancer (Bauer et al. 2010; Kolacinska et al. 2012; Zheng et al. 2014; Le Large et al. 2019). Several lines of evidence suggest functional redundancies between MAP2, tau and other MAPs as well as cytoskeletal regulators (Dehmelt and Halpain 2005).

In summary, we hypothesize that MAP2 might be a candidate biomarker for an improved prognostic evaluation of prostate cancer specimens. To the best of our knowledge, MAP2 expression has not been investigated in this context before.

## Materials and methods

### Patient cohort

In total, 310 patient samples were included in the study and recruited from the archives of the Institute of Pathology, University Bonn. All patients suffered from PCA and had undergone radical retropubic prostatectomy in the Department of Urology (University Hospital Bonn) between 1998 and 2008. In each case, PCA was affirmed by routine diagnostic procedures by experienced pathologists. As the samples were recruited over a longer retrospective period of time the primary diagnostics were based on different variants of Gleason Grading. Therefore, we performed a re-evaluation at the time of the TMA construction to ensure consistent Gleason Grading at a given time point. The grading underlying this study corresponds to the ISUP consensus criteria from the year 2005. To minimize inter-observer variability, we formed a consensus between two investigators who were entrusted with the TMA construction. The clinicopathological characteristics are summarized in Table 1. The mean age at diagnosis and follow-up characteristics of our cohort were in accordance with the published epidemiologic distribution and clinical course.

**Table 1:** Clinicopathological parameters (TMA cohort)

**Age (years)**	
Mean	64.31
Standard deviation	6.078
Min–max	45–83
	n
**pT status**	
pT2	201
pT3	105
pT4	4
**N status**	
pN0	282
pN1	24
**ISUP grade group**	
ISUP 1	108
ISUP 2	82
ISUP 3	62
ISUP 4	28
ISUP 5	30
**PSA**	
< 10 ng/ml	202
> 10 ng/ml	90
BCR	
Mean follow-up (months)	71.8
**BCR**	61
No BCR	210

PSA unknown n: 18, pN status unknown n: 4, BCR status unknown n: 39

In total, 211 patient sera were collected between 2018 and 2020 for the measurement of MAP2 serum levels using an enzyme-linked immunosorbent assay (ELISA). 173 blood samples from histologically confirmed PCA (126 samples) and BPH (47 samples) were provided by the Department of Urology (University Hospital Bonn) from patients who underwent regular follow-up examinations. Furthermore, 19 PCA samples were provided by the Department of Nuclear Medicine (University Hospital Bonn). The normal controls were 19 serum samples from apparently healthy volunteers (age 23 to 57 years) with MAP2 levels ranging from 0.0 pg/ml to 46.6 pg/ml (mean 20.6 pg/ml). There was no correlation between age and MAP2 serum levels (R^2^ = 0.0088).

PCA and BPH diagnoses were confirmed by routine diagnostic procedures by experienced pathologists at the Institute of Pathology, University Bonn. The mean age at the date of blood sampling was 70 years for PCA patients, 65 years for BPH and 32 years for control patients.

### Tissue microarray construction

From the above-mentioned series we established and investigated a TMA cohort (*n* = 310). Tissue specimens were analysed using TMAs comprising 5 tissue samples of 1.2 mm diameter for each case. The TMAs included benign prostate tissue, prostatic intraepithelial neoplasia (PIN) and invasive carcinoma. Coexisting PIN lesions were included in the tissue microarray (TMA) cohort in 226 cases.

### Immunohistochemistry (IHC)

The paraffin-embedded TMAs were freshly cut (3 µm) and mounted to Super Frost Plus Slides. The sections were pretreated with citrate buffer (pH 6). The staining was performed at the Ventana Benchmark automated staining system (Ventana Medical System, Tuscon, AZ, USA) following the manufacturer’s protocol. In brief, the slides were incubated with a monoclonal MAP2 antibody (dilution: 1:250, clone HM2, mouse IgG1 isotype, Sigma, M4403, USA). The signal was detected using an ultraView Universal DAB detection kit Ventana Medical System, Tuscon, AZ, USA) conjugated with the secondary antibody. Application of Ultra wash system (Ventana) reduced background coloring. Finally, counterstaining was performed with hematoxylin, then the slides were coverslipped.

### Immunohistochemical analyses

MAP2 expression was determined both in whole block sections and in TMA sections. Immunohistochemical stainings were analyzed using an Olympus BX51 microscope and the Panoramic Viewer 3DHistech. Whole block sections of 107 prostatectomy specimens were recruited for descriptive statistics and to test for a possible MAP2 expression heterogeneity in different tumor areas. The staining intensity in invasive PCA was assessed semi-quantitatively for each Gleason pattern (GP) (negative = 0, weak = 1, moderate = 2, strong = 3), based on the predominant staining intensity. In addition, MAP2 expression was assessed with a semi-quantitative adapted immunoreactive score, as described by Remmele and Stegner, multiplying a score for cytoplasmic staining intensity of positive cells (0 = negative, 1 = weak, 2 = moderate, 3 = strong) with the percentage of stained cells (0 = 0, 1 < 10%, 2 = 10–50%, 3 = 51–80%, 4 > 80%)(Remmele and Stegner 1987).

**Fig. 1:**
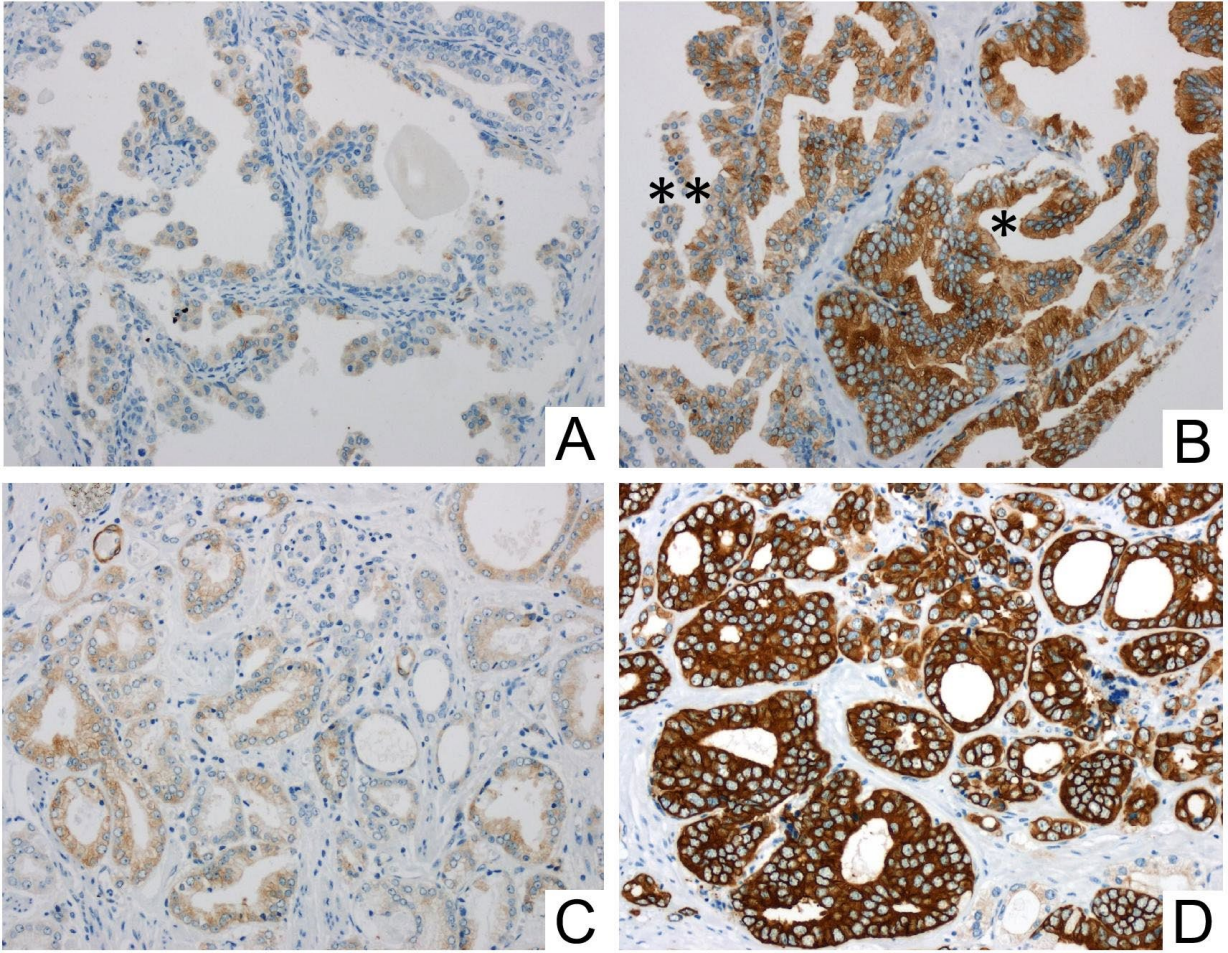
Quantification of immunohistochemical staining using H-scores in exemplary TMA tissue samples: **A** non-neoplastic glands only show low stainability in about 30% of the cells, corresponding to an H-score of 30. **B** H-scores in PIN lesions: the high-grade PIN (*) shows greater stainability (H-score: 270) than the low-grade PIN (**) (H-score: 150). **C** shows invasive carcinoma (GP3) with only mild to moderate expression (20% of the tumor cells without stainability, 70% of the tumor cells with mild stainability and 10% of the tumor cells with moderate stainability, resulting in an H-score of 90. **D** shows a tumor proportion with strong stainability (here using the example of cribriform tumor cells; GP4): 90% of the cells are strongly stained, 10% of the cells show moderate stainability, corresponding to an H-score of 90 × 3 + 10 × 2 = 290. (200× magnification)

In the (TMA) cohort, the IHC was evaluated by H-score as described before by McCarty et al. (McCarty et al. 1985). In brief, the H-scores were calculated by the sum of individual H-scores for each intensity level seen, using the following formula: 1 × (% weakly stained cells) + 2 × (% moderately stained cells) + 3 × (% strongly stained cells), giving a possible range of 0–300. H-scoring is illustrated in Fig. 1. H-scores were determined for each Gleason pattern (GP) observed in a given case. The case-related H-score was then calculated using the equivalent weighting of the GP H-scores, i.e., the GP scores of a given case were equally added and then the sum was divided by the number of GPs included. If a case was only represented by a single GP, the case-related H-score was identical to the GP score. The IHC data of the TMA cohort were correlated with clinicopathological parameters. In addition, a Kaplan—Meier analysis was carried out with regard to BCR-free survival. BCR was defined as PSA relapse ≥ 0,2 ng/ml after prostatectomy. The initial evaluation of the immunohistochemical MAP2 staining in whole block sections revealed cases with heterogeneous staining properties and those with strong and continuous MAP2 expression. In the TMA cohort, these differences in the expression patterns were best delineated by dichotomizing H-score groups lower or higher than approximately 200. Therefore, expression data were dichotomized in the two groups H-score ≤ 200 and H-score ˃200, i.e., weak-expression versus strong-expression.

### Enzyme-linked immunosorbent assay

Serum was obtained by coagulating whole blood samples at room temperature for 20 min and subsequent centrifugation at 3000 rpm for 10 min at 4 °C. The supernatant was immediately aliquoted and frozen at −20 °C. MAP2 measurement was performed via MAP2 ELISA Kit (OKCD02710, Aviva Systems Biology, San Diego, CA, USA) according to the manufacturer’s instructions. Absorbance was measured in triplicates at 450 nm wavelength on an Infinite 200 PRO Microplate Reader (Tecan, Männedorf, Swiss). According to the manufacturer’s instructions, wavelength correction was carried out by subtracting absorbance measured at 540 nm from absorbance measured at 450 nm. This subtraction will correct any optical imperfections that may be present in the plate.

Assays were performed in a 96-well plate format. To determine protein concentrations, a plate-specific standard curve was prepared for each assay. For analysis, the mean of each triplicate was calculated with subsequent subtraction of the blank value (relative OD_450_). MAP2 concentration contained in each sample was interpolated using linear regression of the sample relative OD_450_ against the standard curve. Subsequently, MAP2 levels were presented in either two subgroups (non-PCA and PCA) or three subgroups normal, BPH and PCA. Furthermore, MAP2 levels were compared to the ISUP Grade Groups and blood PSA levels.

### Statistical analyses

All statistics were generated using SPSS version 29.0 (IBM SPSS Chicago, USA). Chi-square tests were performed to evaluate the statistical significance of associations between MAP2 expression and clinicopathological parameters. The non-parametric Kruskal–Wallis test was applied for group comparisons. Univariate survival analyses were calculated using Kaplan–Meier statistics. Log-rank values were used to evaluate differences in survival curves. Multivariate survival analysis was performed for all parameters that were found to be significant in the univariate analysis with the Cox regression model. *p* < 0.05 was considered significant.

For the enzyme-linked immunosorbent assay, data were presented as mea* n* ± standard deviation. Mean serum MAP2 levels were indicated by horizontal bars. Statistical significance was determined by students t-test (two0tailed for independent samples) with *p* < 0.05 considered as significant (*p < 0.05; ** *p* < 0.005; *** *p* < 0.001).

Relationships between the two independent variables (MAP2 and PSA) were determined by linear regression and visualized using a trendline. Goodness of fit was ascertained by the coefficient of determination (R^2^).

## Results

### MAP2 expression in whole block sections

MAP2 expression was determined in whole block sections of 107 prostatectomy specimens. Non-neoplastic prostatic glands were MAP2 negative (*n* = 24) or revealed a weak MAP2 staining intensity (*n* = 80). In three cases, a moderate expression of non-neoplastic prostatic glands was focally observed. Strong MAP2 expression was not found in non-neoplastic prostatic glands. High-grade PIN lesions were found in nearly two thirds of the cases (*n* = 66). Within this subset, weak MAP2 expression was found in 6.1%, moderate expression in 34.8% and strong expression in 57.8% of the cases. One high-grade PIN lesion was MAP2 negative (1.5%). Invasive tumor glands revealed at least focal MAP2 expression in 86% of the cases. MAP2-positive GP 3 tumor glands were found in 86.4%. Within this subset, weak MAP2 expression was found in 13.6%, moderate expression in 24.3% and strong expression in 48.5% of the cases. MAP2-negative tumor glands (GP 3) were found in 13.6% of the cases. MAP2-positive GP 4 tumor glands were found in 77.8%. Within this subset, weak MAP2 expression was found in 22.2%, moderate expression in 27.8% and strong expression in 30.6% of the cases. MAP2-negative tumor glands (GP 4) were found in 19.4% of the cases. MAP2-positive GP 5 tumor glands were found in 75%. Within this subset, moderate MAP2 expression was found in three and strong expression in three of the cases.

**Fig. 2:**
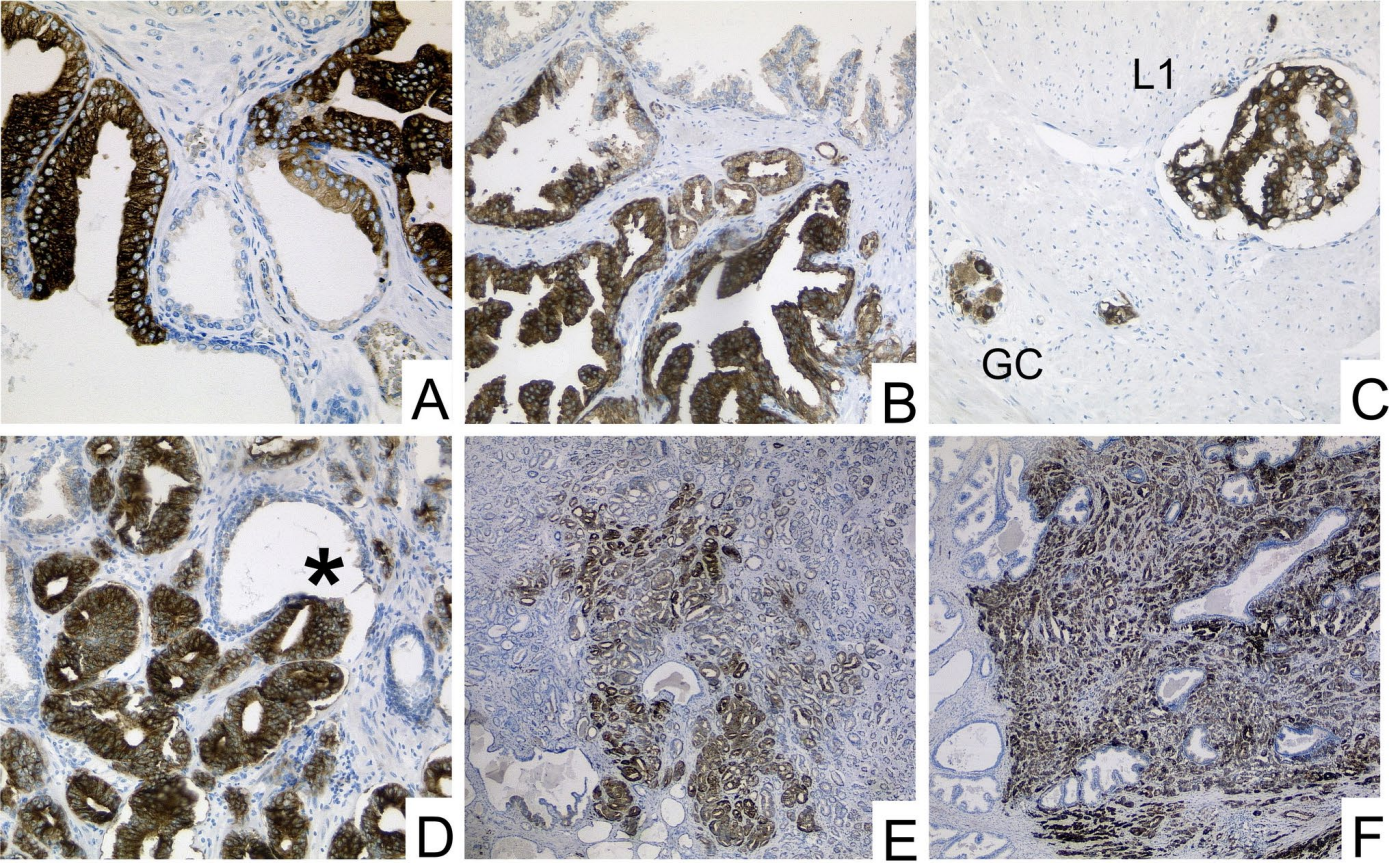
Strong MAP2 labeling in PIN lesions **A** and at the interface between PIN and invasive carcinoma **B**. MAP2 positive staining is also observed in lymphangioinvasive carcinoma cells (L1)—here in close proximity to ganglion cells (GC) **C**. The tumor shows strong staining in the area of invasion into benign glandular tissue (*) **D**. Two main staining patterns of the invasive cells can be observed: proliferation with gradual loss of MAP2 expression leading to a patchy staining pattern (**E**) and cases with uniform MAP2 staining (**F**). (A-D: 200 × magnification, E–F: 100 × magnification)

**Fig. 3:**
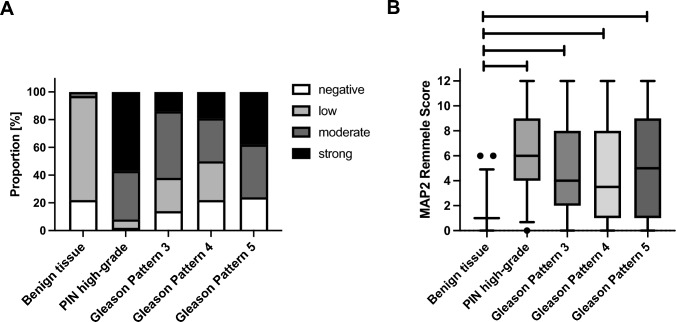
MAP2 immunolabeling in PCA, PIN and benign prostatic tissue (whole block sections, *n* = 107). **A** Relative distribution of MAP2 expression in benign prostatic tissue, PIN and PCA (GP3, GP4, GP5) according to staining intensities in whole block sections of 107 prostatectomy specimens. **B** Illustration of the four-scaled assessment showed significantly stronger MAP2 expression in carcinoma and PIN than in benign tissue. Remmele Scores of MAP2 expression were significantly higher in both high-grade PIN lesions and each invasive Gleason pattern (GP 3, GP 4, GP 5) than in benign prostatic glands. The lowest mean RS was found in non-neoplastic prostatic glands (mean RS 1.2 (standard deviation (SD) 1.1). High-grade PIN lesions had the highest mean RS of 6.1 (SD 3.0) and the RS scores of invasive tumor glands were as follows: GP3: RS 4.6 (SD 3.4); GP4: RS 4.3 (SD 4.1) and GP5: RS 5,5 (SD 4.3). * p < 0.05, **** p < 0.0001. Benign tissue: *n* = 107, high grade PIN: *n* = 66 GP3: *n* = 103, GP4: *n* = 36, GP5: *n* = 8

MAP2 expression was predominantly found at the interface between high-grade PINs and invasive tumor cells. MAP2 immunohistochemistry either revealed expression with a gradual loss of MAP2 expression leading to a patchy staining pattern or it was characterized by a rather uniform MAP2 staining in different tumor areas (Figs. 1 and 2). Due to these staining heterogeneity, we have evaluated according to the Remmele score (RS) (i.e., staining intensity (0–3) multiplied by the percentage of positive cells (0–4)) to determine the expression rate over larger areas. The mean RS of high-grade PIN lesions and invasive GP (GP 3, GP 4, GP 5) were significantly higher than in non-neoplastic prostatic glands (*p* < 0.01). The lowest mean RS was found in non-neoplastic prostatic glands (mean RS 1.2 (standard deviation (SD) 1.1). High-grade PIN lesions had the highest mean RS of 6.1 (SD 3.0) and the RS scores of invasive tumor glands were as follows: GP3: RS 4.6 (SD 3.4); GP4: RS 4.3 (SD 4.1) and GP5: RS 5.5 (SD 4.3) (Fig. 3b).

### MAP2 expression in TMA specimens

**Fig. 4:**
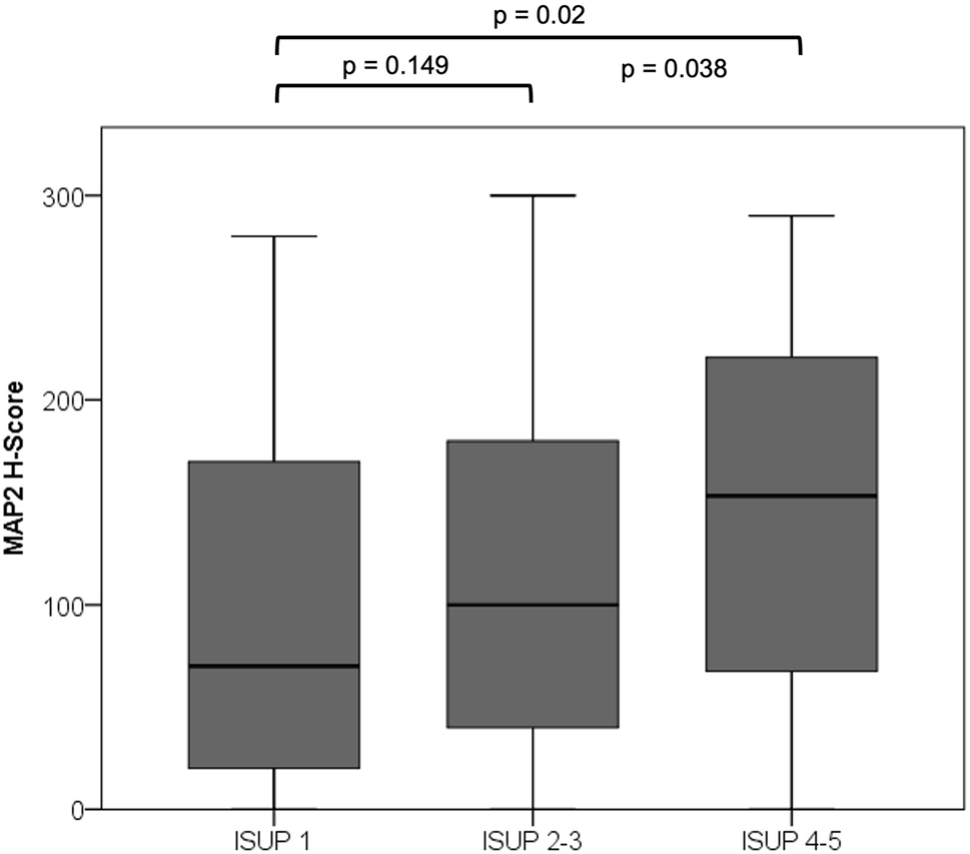
MAP2 expression in PCA (TMA IHC). MAP2 is significantly increased in high-grade carcinomas (ISUP Grade Group 4–5). *n* = 310; ISUP 1: *n* = 108, ISUP 2–3: *n* = 144, ISUP 4–5: *n* = 58

**Fig. 5:**
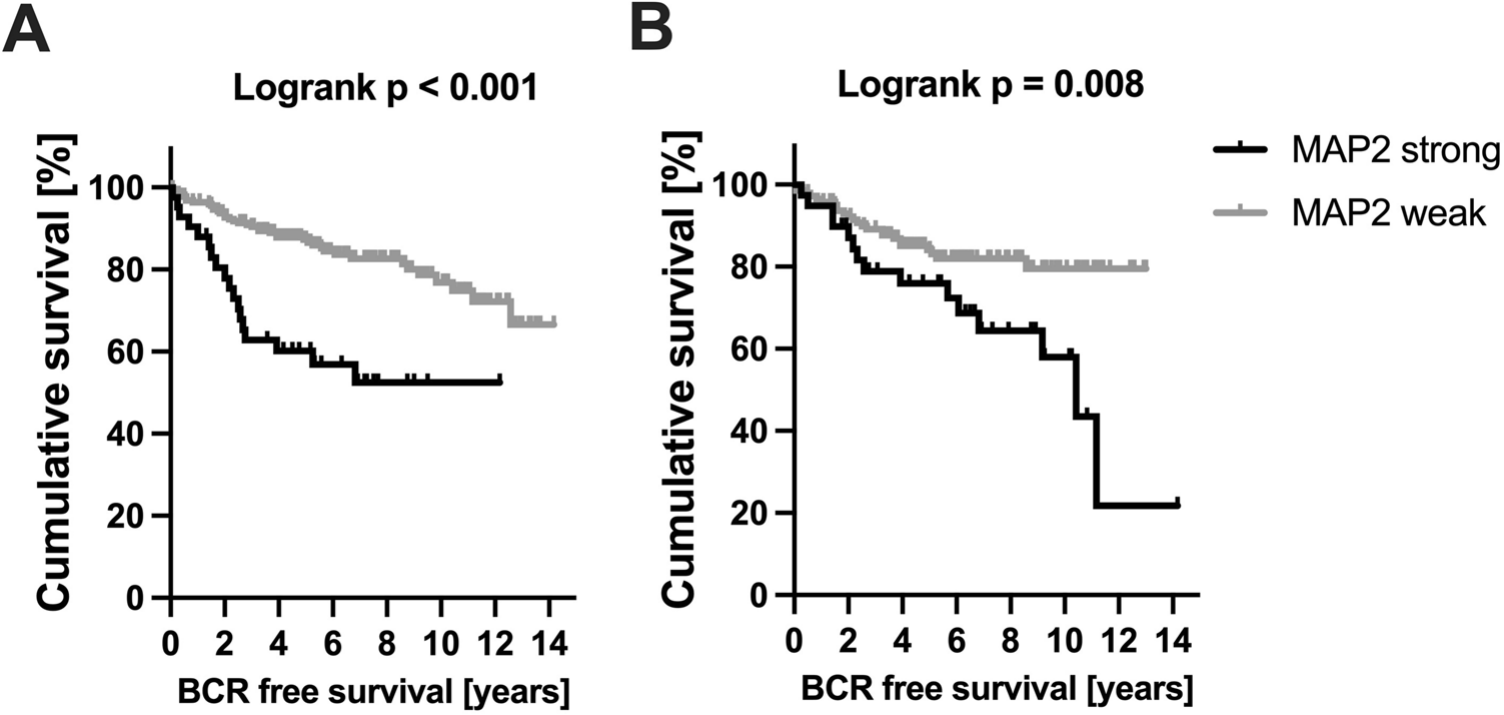
BCR-free survival following prostatectomy stratified by MAP2 expression. Strong MAP2 staining in carcinoma (*n* = 271; MAP2 strong: *n* = 43, MAP2 weak: *n* = 228) **A** and PIN lesions (*n* = 186; MAP2 strong: *n* = 40, MAP2 weak: *n* = 146) **B** is associated with a shortened BCR free survival

A TMA-based analysis, comprising a cohort in 310 cases with established clinicopathological and precise follow-up data was performed (Table 1, 2). MAP2 expression was correlated with the Gleason Score / ISUP Grade Group (Fig. 4). Poorly differentiated carcinomas (ISUP 4–5, *n* = 58) were strongly stained in 63.8%, whereas MAP2 was significantly less expressed in carcinomas with ISUP Grade Group 1–3. The PIN lesions were strongly immunolabeled in 20.8% of the cases. Interestingly, high MAP2 expression in PIN lesions correlated with the presence of simultaneous high-grade carcinoma (ISUP 4–5) (*p* = 0.019) (Table 1, 2). In both carcinoma and PIN lesions, MAP2 staining correlated with the preoperative PSA value. pT-status and lymph node metastasis did not correlate with MAP2 expression (Table 1, 2). Kaplan Meier analysis showed a significant correlation between strong MAP2 staining in carcinoma and shortened BCR-free survival after prostatectomy (*p* < 0.001). Remarkably, high MAP2 expression in PIN was also correlated with a shortened BCR-free survival (*p* = 0.008, Fig. 5).

**Table 2:** MAP2 staining and correlation with clinicopathological parameters (TMA cohort)

Parameter	*n*	MAP2 carcinoma			*n*	MAP2 PIN		
		H-Score ≤ 200	H-Score > 200	*p*-value		H-Score ≤ 200	H-Score > 200	*p*-value
pT status								
pT2	201	169	32	0.585	153	124	29	0.325
≥ pT3	109	89	20		73	55	18	
N status								
pN0	282	233	49	0.51	210	166	44	0.74
pN1	24	21	3		12	9	3	
ISUP grade group								
ISUP 1–3	252	221	31	< 0.001	189	155	34	0.019
ISUP 4–5	58	21	37		37	24	13	
PSA								
< 10 ng/ml	202	174	28	0.015	156	130	26	0.04
> 10 ng/ml	90	67	23		54	38	16	
	PSA unknown n:18 pN status unknown n:4				PSA unknown n:16 pN status unknown n:4

As expected, univariate survival analysis confirmed the parameters pT-status (*p* = 0.001) and Gleason Score/ISUP Grade Group (*p* < 0.001) as significant prognostic parameters. In contrast, preoperative PSA levels and N-status were not prognostically relevant (*p* = 0.536 and *p* = 0.16, respectively). As high MAP2 expression was correlated with grade, we hypothesized that this unequal distribution may contribute to its adverse prognostic effect. However, multivariate Cox regression analysis validated MAP2 as an independent prognostic marker when tested together with ISUP Grade Groups and T-status (Table 3). In contrast, MAP2 expression in PIN lesions lost its significant adverse prognostic effect performing the multivariate analysis.

**Table 3:** Multivariate Cox regression analysis with regard to BCR-free survival after prostatectomy revealed MAP2 immunostaining as an independent prognostic marker

Parameter	*p*-value	Hazard ratio	CI 95%	
ISUP grade groups	< 0.001	1.505	1.232	1.838
pT-status	0.110	1.550	0.906	2.651
MAP2	0.018	2.007	1.127	3.574

**Fig. 6:**
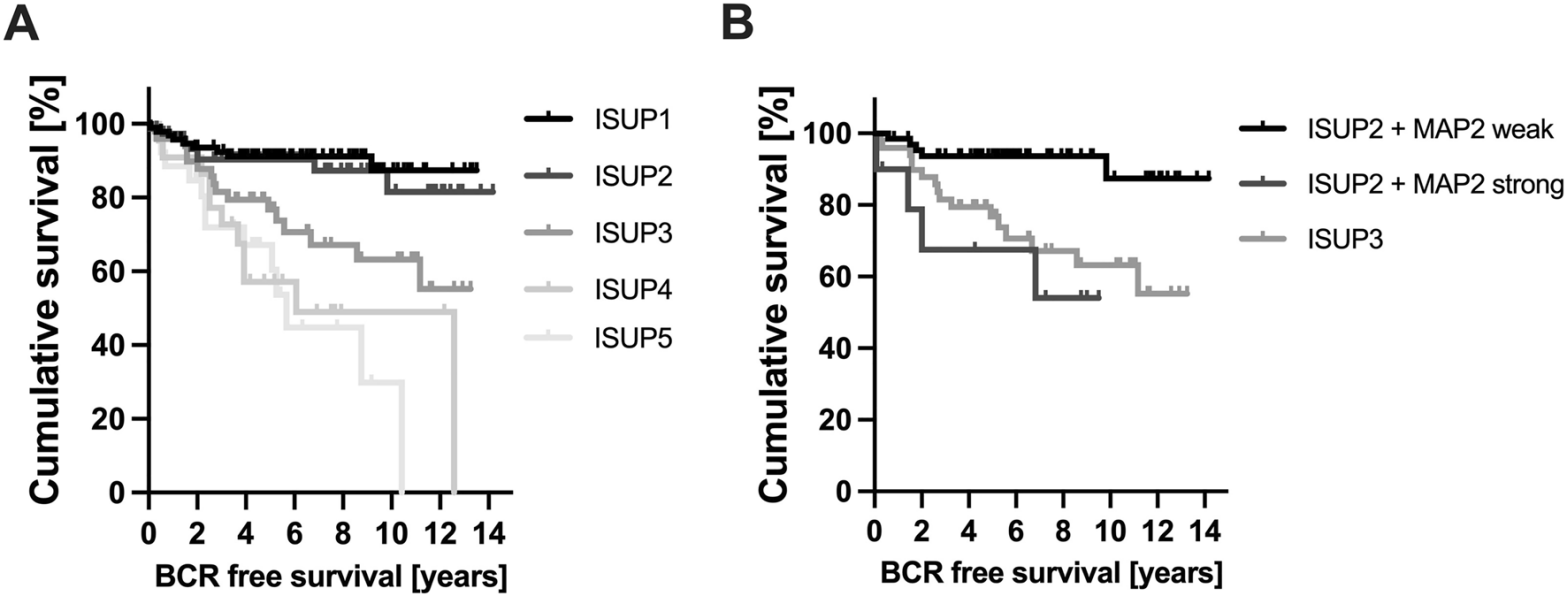
**A** Kaplan Meier curves illustrate BCR-free survival stratified by ISUP Grade Groups (TMA cohort). *n* = 271; ISUP 1: *n* = 94, ISUP 2: *n* = 77, ISUP 3: *n* = 51, ISUP 4: *n* = 23, ISUP 5: *n* = 26. **B** Subgroup analysis of the prognostically heterogeneous ISUP group 2. MAP2 expression data enables subdivision into two prognostic subgroups; ISUP 2 MAP2 weak with extended and ISUP 2 MAP2 strong with shortened BCR free survival similar to ISUP 3 (ISUP 2 MAP2 weak vs. ISUP 2 MAP2 strong, Log Rank *p* = 0.002; ISUP 2 MAP2 strong vs. ISUP 3, Log Rank *p* = 0.538). *n* = 128; ISUP2 MAP2 weak *n* = 66, ISUP2 MAP2 strong *n* = 11; ISUP3 *n* = 51

As illustrated in Fig. 6 the ISUP Grade Group solely was unable to predict a significant difference in BCR-free survival between ISUP Grade Groups 1 vs. 2 (*p* = 0.054), ISUP 2 vs. ISUP 3 (*p* = 0.714) and ISUP 4 vs. 5 (*p* = 0.139) in our cohort (exception: ISUP 3 vs. 4; *p* = 0.01). Interestingly, the MAP2 subgroup analysis for the individual ISUP groups 1–5 revealed statistical significance in the survival analysis for ISUP groups 2 and 5 (Table 4). Comparing the MAP2 stratified ISUP 2 with ISUP 3, the clinical course of the ISUP 2 cases with high MAP2 expression was comparable to those of the ISUP 3 (*p* = 0.538, Fig. 6).

**Table 4:** Subgroup analysis: Correlation of MAP2 expression and BCR in different ISUP Grade Groups

ISUP grade group	*n*	Number of events	Pearsons- × 2	*p*-value
ISUP 1				
H-score ≤ 200	87	9	0.230	0.631
H-score˃200	7	1		
ISUP 2				
H-score ≤ 200	68	6	10.885	0.001
H-score˃200	9	4		
ISUP 3				
H-score ≤ 200	40	12	1.963	0.161
H-score˃200	11	5		
ISUP 4				
H-score ≤ 200	15	9	1.003	0.317
H-score˃200	8	2		
ISUP 5				
H-score ≤ 200	18	7	5.120	0.024
H-score˃200	8	6		

### MAP2 levels in patient serum

**Fig. 7:**
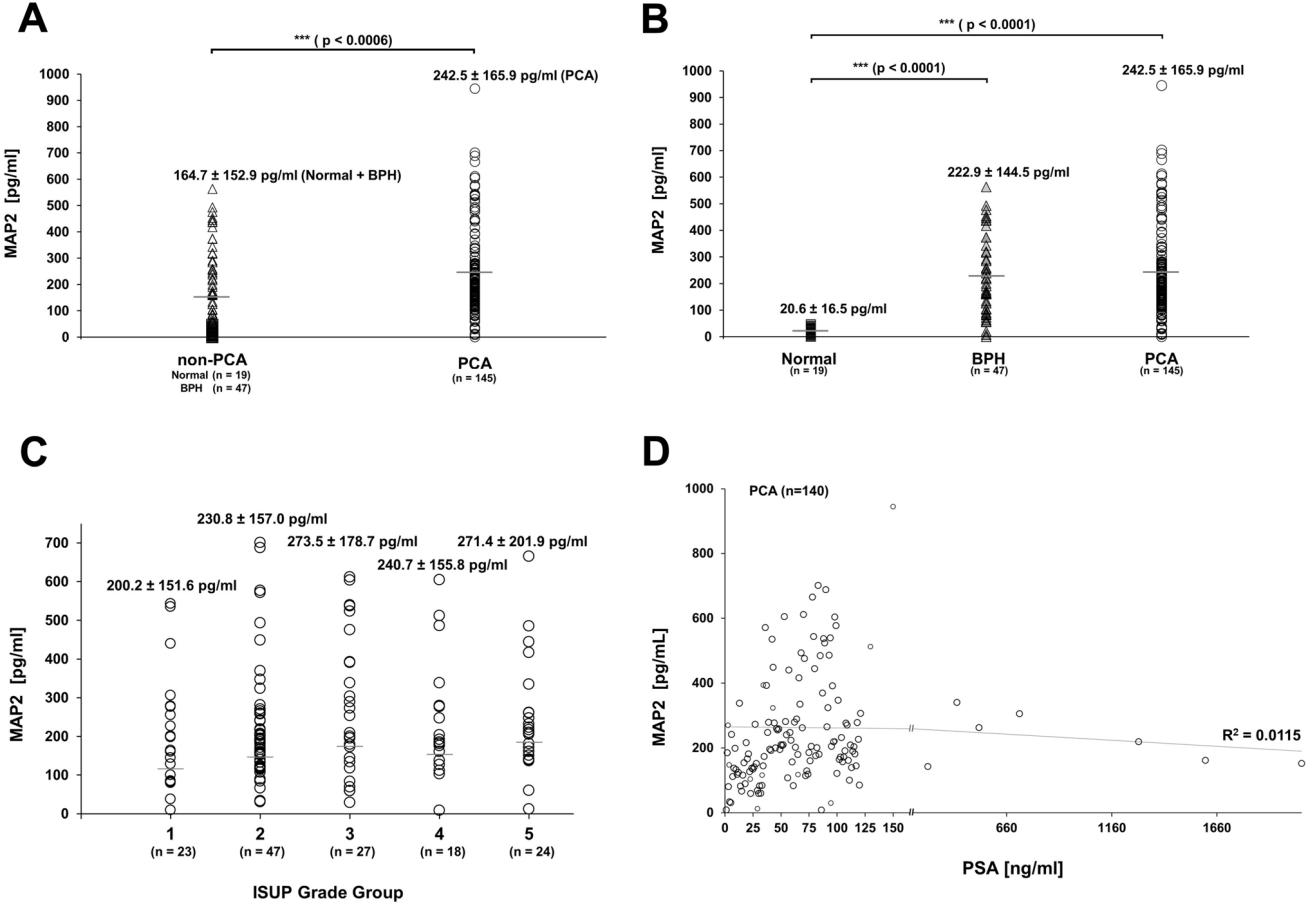
MAP2 serum levels in healthy controls, BPH and PCA patients. **A** Comparison of MAP2 levels in two subgroups: non-PCA vs. PCA, *** = * p* < 0.0006. **B** Comparison of MAP2 levels in three subgroups: normal, BPH and PCA, *** = * p* < 0.0001. **C** Serum MAP2 levels vs. ISUP grade groups 1–5. MAP2 levels are presented numerically as mea* n* ± standard deviation. Mean MAP2 levels are indicated by grey horizontal bars (**A**–**C**). **D** MAP2 levels of PCA patients vs. PSA serum levels. A trend line and the coefficient of determination (*R*^2^ = 0.0115) describe the correlation of MAP2 and PSA

Detection of MAP2 protein levels in serum was performed by ELISA. In total, 145 PCA and 66 non-PCA patient samples were analysed for their MAP2 levels in serum. Interestingly, PCA samples (mean MAP2 of 242.5 pg/ml) showed significantly increased MAP2 levels compared to non-PCA samples (mean MAP2 level = 164.7 pg/ml, *p* < 0.0006) (Fig. 7A). A more precise analysis revealed a highly statistically significant difference between normal vs BPH and normal vs PCA, *p* < 0.0001 and *p* < 0.0001, respectively (Fig. 7B). Although mean serum MAP2 levels were slightly higher in PCA than in BPH, the difference did not reach statistical significance.

In addition, MAP2 levels did not differ significantly between the different ISUP Grade Groups. However, a modest trend between MAP2 protein levels and increasing ISUP Grade Groups was observed (Fig. 7C). There was no correlation between MAP2 and PSA serum levels of PCA patients, as shown in Fig. 7D.

## Discussion

This study is the first to demonstrate overexpression of MAP2 in the majority of PCA cases compared to non-neoplastic prostatic glands. It also contributes further evidence that MAP2 expression is not limited to the central nervous system (CNS) and neuroendocrine tumors but is also found in solid non-neuronal tumors.

The immunohistochemical data were furthermore supported by the measurement of MAP2 protein levels in serum. These analyses showed significantly increased PCA MAP2 serum levels (mean 242.5 pg/mL) as compared to normal / BPH samples (mean 149.5 pg/mL, *p* < 0.001). Although MAP2 serum levels slightly increased with higher ISUP Grading, analyses did not yield statistical significance (*p* > 0.05). Moreover, there was no significant correlation between MAP2 and PSA serum levels. To further elucidate whether MAP2 protein level in serum could have clinical relevance as a biomarker, further studies have to analyse the course of MAP2 protein levels after local therapy and in the follow-up.

Aberrant expression of the closely related MAPT has been recently observed as an independent prognostic feature in prostate cancer. MAPT was absent in normal prostate epithelial cells but detectable in a proportion of PCA cases. Furthermore, MAPT expression was associated with high Gleason Grade, high T stage and BCR (Schroeder et al. 2019; Sekino et al. 2020). In addition, MAPT expression has been shown to be significantly associated with poor overall survival in patients who were treated with androgen deprivation therapy (Sekino et al. 2020). The phosphorylation status of MAPT is believed to be a key marker for G2/M phase in prostate cancer cells and the forced modulation of Tau phosphorylation can interfere with the capacity of the cell to efficiently progress through G2/M phase, indicating a functional relevance of microtubul-associated proteins in the PCA disease process (Clementi et al. 2023). We also found heterogeneous MAP2 expression in the majority of cases and many MAP2-positive TMA spots revealed both MAP2-positive and MAP2-negative cancer areas. The heterogeneous distribution pattern, which is very similar to MAPT, may indicate a co-localization and functional association of the different MAP proteins. MAPT and MAP2 double staining may be an interesting approach for further immunohistochemical investigations.

From a methodological point of view, heterogeneity raises concerns about the sensitivity and specificity of immunohistochemical investigations. Based on two cohorts, MAPT expression was reported to be positive in 23% and 8% of cases, respectively (Cirak et al. 2013; Schroeder et al. 2019). Therefore, we generally agree with the assessment of Schroeder et al., that such expression heterogeneity represents a limitation for TMA studies analyzing only rather small tissue areas per tumor. However, in contrast to the MAPT findings mentioned before, at least focal MAP2 expression could be detected in 79% of the TMA specimens (H-score cut off > 20), so the risk of false-negative findings in our TMA cohort is rather lower despite expression heterogeneity. In addition, histopathological examinations of the whole block sections revealed rather a small-scale expression heterogeneity with positive and negative areas close to one another, thereby raising the detection probability of MAP2-positive tumor areas even in the TMA setting.

From a pathogenetic point of view, the predominant expression of MAP proteins in neoplastic tissue may be an indicator of cytoskeletal remodelling processes during carcinogenesis. MAP2 was pronouncedly expressed both in high-grade PIN lesions and in adjacent invasive tumor areas. These findings may lead to the hypothesis, that cytoskeletal remodelling processes including microtubule-associated proteins may play a crucial role in the transition of in-situ cells into invasive cancer cells. Our findings suggest an upregulation of MAP2 during carcinogenesis and provide MAP2 as a promising tool for histopathological diagnostic procedures. The assessment of a MAP2 expression profile might add valuable prognostic information for risk assessment in a pre-surgical diagnostic setting. However, additional studies are needed that focus on the histopathological findings in prostate biopsies before radical prostatectomy. Immunohistochemical data were supported by the measurement of MAP2 protein levels in serum. These analyses showed significantly increased PCA MAP2 serum levels as compared to normal samples. The same increase was also observed when comparing normal samples to BPH MAP2 serum levels. The significance of these findings, however, might be affected by the substantial age difference between normal and BPH/ PCA samples in this cohort. There was no significant correlation between MAP2 and PSA serum levels. Although MAP2 serum levels slightly increased with higher ISUP Grade Groups, analyses did not reach statistical significance.

The evaluation of MAP2 expression may help to further delineate the ISUP Grade Groups. The present data showed that the clinical course of the ISUP Grade Group 2 cases with strong MAP2 expression is significantly different from those with only weak to moderate MAP2 expression (ISUP 2 MAP2 weak vs. ISUP 2 MAP2 strong, *p* = 0.002). And vice versa, ISUP Grade Group 2 cases with strong MAP2 expression are comparable to that of ISUP Grade Group 3 concerning the clinical course (Fig. 5b). This is of special clinical interest as ISUP 2 tumors (< 10% GP 4) may be considered for active surveillance (N.Mottet et al. 2023). Therefore, our findings may open new avenues towards a more sophisticated tumor classification by modifying classical haematoxylin–eosin (HE) based tumor classification procedures by immunohistochemical parameters such as expression of MAP2. To further elucidate whether MAP2 protein level in serum could have clinical relevance as a biomarker, further studies have to analyse the course of MAP2 protein levels after local therapy and in the follow-up. In this context, high expression levels of MAP2, as a risk-modifying factor, could also influence therapy decisions in the future.

From a therapeutic point of view, MAP2 may also represent an interesting target protein. High MAP2 expression correlated with paclitaxel and docetaxel sensitivity in pancreatic carcinoma cell lines (Veitia et al. 2000; Le Large et al. 2019). Moreover, MAP2 expression in breast cancer specimens was correlated with increased sensitivity for paclitaxel (Bauer et al. 2010; Kolacinska et al. 2012). Taxanes are microtubule-stabilizing cytotoxic agents (MSAs) that convey a survival benefit in patients with metastatic prostate cancer. Independent of promoting mitotic arrest, MSAs can suppress the nuclear accumulation of androgen receptor, which is the driving force for prostate cancer cell growth and progression. These data suggest that the functional status and activity of the microtubule system influence the therapeutic benefit of chemotherapeutic approaches(Chen 2023).

The taxanes docetaxel and cabazitaxel are recommended chemotherapeutic agents for metastatic prostate cancer according to the current guidelines(N.Mottet et al. 2023). Future studies investigating the association of MAP2 expression and response to chemotherapy in the metastatic stage are therefore promising and necessary.

The present immunohistochemical data were obtained using prostatectomy specimens, so it is unclear whether the results and the interpretations can be transferred to the preoperative situation, especially in the context of punch biopsies. An analogous evaluation of immunohistochemical MAP2 expression in punch biopsy specimens may provide further information about whether MAP2 expression may be a suitable biomarker in the context of preoperative diagnostics and therapy planning procedures. Future studies are necessary to address this critical issue.

In summary, MAP2 expression is frequently expressed in PCA, is correlated with grade in TMAs, and is associated with an increased risk of BCR after radical prostatectomy. The present findings raise the question of whether the evaluation of MAP2 expression could also have a diagnostic and prognostic value in preoperative PCA diagnostic procedures, i.e., the evaluation of MAP2 expression in core biopsies, in particular concerning treatment planning. In this context, the dichotomization of ISUP Grade Group 2 (Gleason Score 7a) according to MAP2 expression may be of particular interest concerning patient selection, especially for the treatment option active surveillance.

## Data Availability

The datasets used and analyzed during the current study are available from the corresponding author upon reasonable request.
